# Amyloid fragments and their toxicity on neural cells

**DOI:** 10.1093/rb/rbz007

**Published:** 2019-03-11

**Authors:** Eva Bystrenova, Zuzana Bednarikova, Marianna Barbalinardo, Cristiano Albonetti, Francesco Valle, Zuzana Gazova

**Affiliations:** 1 Institute of Nanostructured Materials I.S.M.N – C.N.R., via Gobetti, 101, Bologna, Italy; 2 Department of Biophysics, Institute of Experimental Physics SAS, Watsonova 47, Kosice, Slovakia

**Keywords:** amyloid fibrils, protein aggregation, neural cells, atomic force microscopy, fluorescence microscopy

## Abstract

The formation of amyloid fibrils from soluble proteins is a common form of self-assembly phenomenon that has fundamental connections with biological functions and human diseases. Lysozyme was converted from its soluble native state into highly organized amyloid fibrils. Ultrasonic treatment was used to break amyloid fibrils to fibrillar fragments–seeds. Atomic force microscopy and fluorescence microscopy was employed to characterize the morphology of the amyloid assemblies and neural cells–amyloid complexes. Our results demonstrate that prefibrillar intermediated and their mixture with proteins exhibit toxicity, although native proteins and fibrils appear to have no effect on number of cells. Our findings confirm that innocuous hen lysozyme can be engineered to produce both cytotoxic fibrillar fragments and non-toxic mature amyloid fibrils. Our work further strengthens the claim that amyloid conformation, and not the identity of the protein, is key to cellular toxicity and the underlying specific cell death mechanism.

## Background 

Cellular systems maintain the balance between protein synthesis and degradation via the quality-control machinery that prevents deposition of partially folded, misfolded or degraded protein in the cells [1]. A misregulation of these systems like change in cellular environment, genetic mutations or ageing can lead to various human diseases such as neurological disorders and various systematic amyloidosis [2]. These degenerative diseases are characterized by the formation of amyloid deposits in different tissues and organs, resulting from uncontrolled self-aggregation of misfolded proteins [3].

Amyloidoses are slow-progressing diseases resulting from a gain of function associated with partially un/folded forms of the proteins. More than 50 different proteins and peptides (Aβ-peptide, tau, α-synuclein, huntingtin, amylin, β2-microglobulin, lysozyme, etc.) have been found to form amyloid aggregates in humans, as intra- or extracellular depositions. A number of studies have supported the view that early prefibrillar aggregates play a role of direct initiators of cellular damage and death [4].

In general, the process of intramolecular protein–protein interactions of polypeptides leading to amyloid fibrillization, depends on two factors: (i) intrinsic properties of protein molecules such as net charge, hydrophobicity of their surfaces, conformational stability and intrinsic propensity of protein sequence (amyloidogenic sequence) to form intermolecular nuclei necessary for aggregation and (ii) environmental factors such as pH, temperature, type and concentration of salts and the presence of additives [5].

By taking advantage of this generic property of proteins, investigation of amyloid fibrillation using non-disease associated proteins such as hen egg white lysozyme (HEWL) can help in deciphering the molecular mechanisms of amyloid fibrilogenesis. HEWL is an archetypal protein widely used to study the mechanisms of protein folding, misfolding and amyloid formation [6]. In this work, we focus on the role that different fibrillar structures have on the viability of neuroblastoma SH-SY5Y cell line.

## Methods

HEWL (L6876), human neuroblastoma SH-SY5Y cells (ECACC 94030304) Thioflavin T (ThT), Dulbecco’s modified Eagle’s medium/Nutrient Mixture F-12 Ham (DMEM F-12) and Triton X100 were purchased from Sigma-Aldrich (St. Louis, MO, USA). Actin Cytoskeleton and Focal Adhesion Staining kit from FAK100, Millipore, USA.

### Amyloid aggregation of lysozyme

HEWL protein (10 mg powder) was dissolved in 1 ml buffer (70 mM glycine, 80 mM NaCl, pH 2.7) to a final concentration 680 µM. This solution was then further diluted to final concentration of 10 μM (147 μg/ml) and lysozyme amyloid fibrils were formed after 2 h incubation at 65°C under constant stirring 1200 rpm.

### Preparation of fibrillar fragments–seeds

Ultrasonic treatment (UST) is often used for preparation of fibril seeds in studies of amyloid aggregation. It breaks fibrils into shorter pieces [7] increasing the number of fibril ends that act as active aggregation sites and accelerating elongation rate without a change of total protein concentration. Fragments of fibrils were obtained via sonication of mature fibril samples. The protocol involved sonication of the fibrils (in phosphate-buffered saline (PBS), refrigerated by immersion in an ice bath) with a tip sonicator (Sonics Vibracell VCX-750) equipped with a 3 mm tapered tip operated at 20% (150 W) power output. Total sonication time was 120 s, obtained with 24 successive 5 s sonication pulses alternated with 5 s pauses. This procedure caused a thorough fragmentation of fibrils into small fibrillar aggregates–seeds.

### ThT fluorescence assay

Presence of amyloid fibrils was assessed by the enhancement of the ThT fluorescence intensity. ThT was added to the samples at a final concentration of 20 μM. Measurements were performed using spectrofluorimeter SynergyMx (BioTek Company, USA) in black 96-well plates. The excitation was set at 440 nm and the emission recorded at 485 nm. The slits were adjusted to 9.0/9.0 nm for the excitation and emission accordingly. All measurements were performed in triplicate and final value represents average of these three-independent measurements.

### AFM measurements

Samples of lysozyme assemblies for atomic force microscopy (AFM) were prepared by drop casting of solution, an aliquot of 10 μl protein solution diluted to a final concentration of 10 μM (147 μg/ml) was deposited on the surface of freshly cleaved mica and after 10 min adsorption the substrate was rinsed with ultrapure water and dried with N_2_ gas. AFM images were taken in the SPM@ISMN facility using two different AFM. For amyloid assembly imaging was used a stand-alone SMENA NT-MDT (NT-MDT, PRA.MA. Sondalo, Italy) microscope operated in semi-contact mode and equipped silicon cantilevers (NSG10, NT-MDT, PRA.MA. Sondalo, Italy) with typical force constant 11.8 N/m. The topographic images were corrected line by line for background trend effect with second-order polynomial fitting (Image Analysis 2.2.0, NT-MDT, PRA.MA. Sondalo, Italy).

AFM images of the neural cells, were taken after immunofluorescent staining and fixing with paraformaldehyde on microscope circular cover glass, on what cells were cultured for 24/48 h. Cover glass was rinsed with ultrapure water and dried with N_2_ gas prior to scan. The AFM imaging of cells–amyloid assembly was performed by Bruker Scanning Probe Microscope Multimode 8 in PeakForce Tapping mode with Sharp Nitride Levers (SNL 10, Bruker Italia, Milano) with nominal spring constant 0.35 N/m. The topographic images were corrected by levelling and data correction, filtering or grain marking functions using Gwyddion: an open-source software for SPM data analysis [8].

### Cell culturing and counting

Human neuroblastoma SH-SY5Y cells (ECACC 94030304; Sigma Aldrich, St. Louis, MO, USA) were cultured in Dulbecco’s modified Eagle’s medium/Nutrient Mixture F-12 Ham (DMEM F-12) with 2 mM l-glutamine, 100 IU/ml penicillin, 100 µg/ml streptomycin and supplemented 15% fetal bovine serum (FBS). Cells were maintained in standard conditions at 37°C in humidified atmosphere (95% humidity, 5% CO_2_) and split regularly (rate 1:10) by trypsinization. Cells (3th–20th passage) were cultured in Petri dish until sub-confluence and then harvested for cell tests. SH-SY5Y cells were seeded (2 × 10^4^ cells/ml, 0.5 ml/cm^2^, 2 × 10^4^ cells/cm^2^) and cultured on glass samples in 24-well plates, for 24–48 h under standard cell culture conditions. For adhesion experiments, SH-SY5Y cells were incubated for 3 h in standard conditions. In all experiments, glass cover-slips were used. The number of neural SH-SY5Y cells after incubation with different amyloid forms was assessed by counting of 4′,6-diamidino-2-phenylindole (DAPI)-stained nuclei using ImageJ software [9].

### Immunocytochemistry

For fluorescence immunocytochemistry, SH-SY5Y cells, grown on glass surfaces for 24 and 48 h, each sample was carefully placed in a new 24 multi-well plate to eliminate any contribution of remnant cells from the cell suspension. Cells were fixed with 4% paraformaldehyde in DPBS 1×, washed with DPBS 1×. They were then permeabilized with 0.01% Triton-X 100. The cells were labelled with tetramethylrhodamine (TRITC)-conjugated phalloidin for map local orientation of actin filaments followed by rinses with DPBS 1×. Nuclear counterstaining was performed by incubation with DAPI for 3 min, followed by rinses with DPBS 1×, and fibrillar aggregates by thioflavin T.

Samples were examined using a Nikon Eclipse 80i microscope equipped for fluorescence analysis.

The blue-fluorescent DAPI, nucleic acid stain, preferentially stains dsDNA; it appears to associate with A-T clusters in the minor groove and allow easy visualization of the nucleus of cell. Binding of DAPI to dsDNA produces a ∼20-fold fluorescence enhancement, apparently due to the displacement of water molecules from both DAPI and the minor groove. The excitation maximum for DAPI bound to dsDNA is 358 nm, and the emission maximum is 461 nm.

TRITC is a bright orange-fluorescent dye with excitation ideally suited to the 532 nm laser line. It is commonly conjugated to antibodies and proteins for cellular imaging applications, since it selectively label F-actin.

ThT is a benzothiazole dye that exhibits enhanced fluorescence upon binding to amyloid fibrils and is commonly used to visualize and quantify the presence of protein amyloid fibrils.

## Results

The aim of this work is to investigate the effect of different well-controlled amyloid aggregates, forming during aggregation process on neural cells growth.

**Figure 1:**
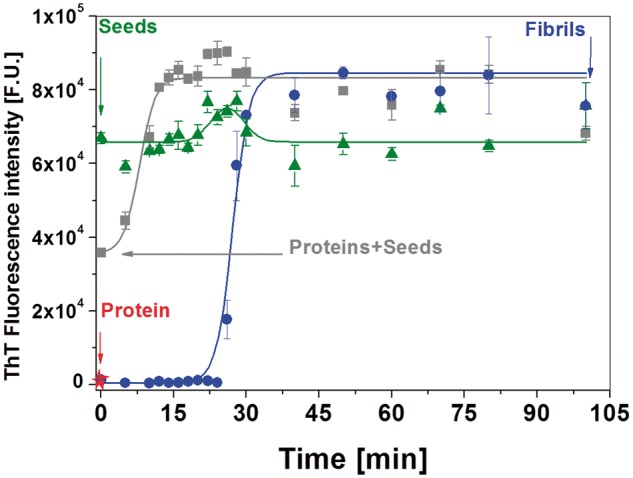
The kinetic curve of lysozyme fibrilization at pH 2.7 and 65°C in 70 mM glycine buffer and 80 mM NaCl. The increase in fluorescence followed sigmoid shape typical of amyloid formation (blue line fit). There is a distinct lag phase of 25 min followed by a gradual increase in ThT fluorescence, indicative of fibril elongation. ThT fluorescence reached a maximum after 30 min of incubation and then remained constant. Green curve shows behaviour of mixture of protein and seeds at the same conditions. In this case fibrils elongation is accelerating and appear after 15 min. Arrows indicate at which point sample for farther experiment was taken

**Figure 2:**
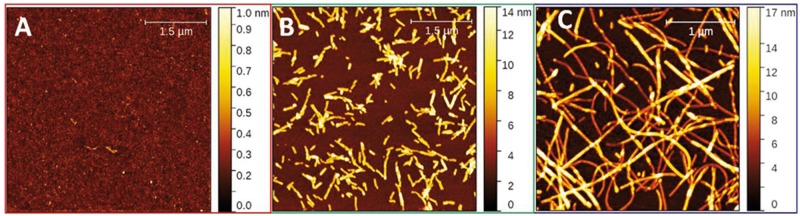
AFM images of hen lysozyme amyloid forms used in neural cells viability experiments: (**A**) freshly dissolved lysozyme, prefibrillar assembly, (**B**) seeds and (**C**) amyloid fibrils samples produced after 2 h of incubation at 65°C and pH 2.7. Seeds of 200 nm and mature fibrils of different lengths from 600 to 2500 nm are observed

Incubation of HEWL under fibrillization triggering conditions (pH ∼2.7, 65°C and constant agitation) led to the formation of amyloid fibrils. The kinetics of amyloid formation was monitored by ThT fluorescence, this dye binds to the cross-β-sheets-containing amyloid, leadings to an increase in its fluorescence intensity (Fig. 1, blue circles). A typical sigmoidal kinetics of fibrils formation was observed with lag phase of 25 min followed by steep increase in fluorescence intensity (elongation phase) and saturation phase reached after 35 min. These phases correspond to formation of nuclei, oligomerization, fibrils elongation and maturation, respectively. Mature fibrils after 2 h incubation exhibited a typical amyloid morphology characterized by long unbranched fibrils with length of 600–2500 nm and diameter of 31 ± 4 nm (Fig. 2C, Table 1) as imaged by AFM and measured by Fiber App software [10].

**Table 1:** Typical geometrical factors of AFM images taken on amyloid assemblies, as shown in Fig. 2

	Seeds	Fibrils
Roughness [nm]	0.31 ± 0.05	0.48 ± 0.07
Length [nm]	180 ± 100	600 – 2500
Width [nm]	48 ±8	31 ± 4
Height [nm]	5 ± 1	6 ± 2

RMS roughness of freshly dissolved proteins is ∼0.06 nm, values for seeds and fibrils are reported.

Although ultrasonication is widely used in medicine, industry and research the effects of sonication on protein assembly and its cytotoxicity remain poorly characterized [11]. UST can induce the monomeric solution of amyloidogenic proteins to form amyloid fibrils [12]. However, ultrasonication can also break down preformed fibrils into uniform fragments [13]. We used this approach to produce a sample of aggregates controlled in size and dispersion, that in the following will be named seeds, as shown in Fig. 2B. These seeds have a uniform length up to 10 times shorter than fibrils 180 ± 100 nm (Table 1).

Short fibrils decomposed by UST can be reconstructed into mature fibrils with the addition of monomeric protein; the regrowth of short fibrils rarely occurs in the absence of monomeric protein [14]. Therefore prepared amyloid seeds diluted into solution of freshly prepared native lysozyme were investigated as well. The kinetics of this process can be described as nucleation-dependent [15]. Authors suggest that added seeds act as catalytic sites that can induce conformation changes in native protein. We supported this hypothesis by showing that it allows the system to bypass the slow nucleation phase, shortens lag phase and reach the growth phase much faster and earlier, growth curve reached plateau after 15 min as shown in Fig. 1 (grey squares). To confirm the aforementioned claim, we run kinetic curve as well for seeds only (Fig. 1, green triangles). It shows short lag phase followed by slight increase in fluorescent signal at 26 min (Gaussian fit line) and reached constant value after 35 min. This could be simple reasoned by dissolving and separating small bundles, created by couple of seeds mechanically attached together. However, it does not reach level of mature fibrils (only 75% of signal) as in the case of presents of proteins in solution with seeds, it clearly indicates, not sufficient number of cross-β-sheet-containing amyloid and elongating fibrils.

Typical AFM images of different amyloid forms used in our study are shown in Fig. 2A) freshly dissolved proteins, B) seeds and C) mature fibrils. Using AFM grain analysis we observed typical aggregated particles roughness of above 0.06 nm of freshly prepared proteins, value for seeds is 0.31 ± 0.05 nm and for mature fibrils 0.48 ± 0.07 nm as reported in Table 1. Further analysis of geometrical parameters was performed for seeds and fibrils only, values reported in Table 1 shows no significant differences except for length of assemblies, where mature fibrils shows varying length from 600 nm to 2500 nm, instead prepared seeds have very define value of 180 ± 100 nm.

**Figure 3:**
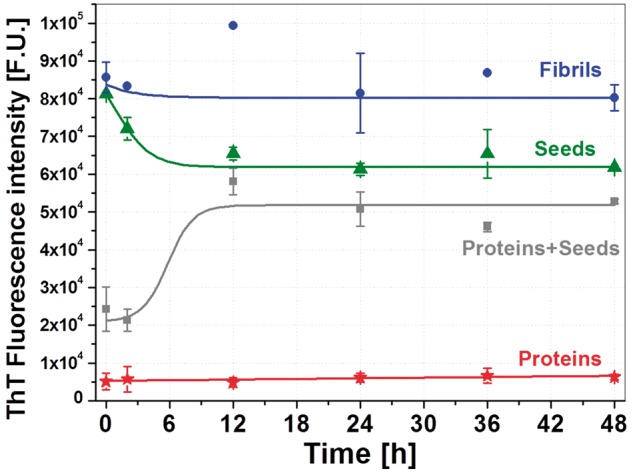
The kinetic curve of all lysozyme assembly used in our study at physiological conditions (pH 7.4 and 37°C in DMEM medium) for 48 h. While proteins (red stars) are stable and do not show any changes all the measure time. Mixture of proteins and seed (green triangles) has a distinct lag phase of 2–3 h followed by a gradual increase in ThT fluorescence reached a maximum after 12 h of incubation and then remained constant, what confirm nucleation growth and elongation of fibrils fragments. The kinetic curve of mature fibrils (blue squares) has similar tendency and timing in elongation of fibrils even if in smaller range. Different behaviour is observed in the case of seeds (grey squares) where for first 8–10 h decrease in ThT signal is observed, what would confirm disassembly of fragments. After 12 h the fluorescence intensities reached steady‐state value

Since the scope of this work was to use controlled assembly of amyloids in cell culture, the prepared samples were incubated under cell growth conditions and their assembly was followed. They were transferred and incubated in the cell-culture medium using ThT assay as presented in Fig. 3 up to 48 h.

The proteins (Fig. 3, red stars) are stable and do not show any changes during measured time. Mixture of proteins and seeds (Fig. 3, grey squares) has a distinct lag phase of 2–3 h followed by a gradual increase in ThT fluorescence. It reaches a maximum after 12 h of incubation and then it remains constant. This confirms nucleation growth and elongation of fibrils fragments. If we take in consideration only first 12 h of kinetic curves, mature fibrils (Fig. 3, blue circles) has similar tendency and timing in elongation of fibrils even if in smaller range. Different behaviour is observed in the case of seeds (Fig. 3, green triangles) where for first 8–10 h the mild decrease in ThT signal is observed, what would confirm disassembly of fragments. This phenomenon could be simply reasoned by dissolving and separating small bundles, created by couple of seeds mechanically attached together. After 12 h the fluorescence intensities reached steady-state value. These measurements were crucial for understanding amyloid assembly behaviour in cell medium compared the condition used in standard aggregation assays. DMEM/F-12 cell culture medium we used is a 1:1 mixture of DMEM and Ham’s Nutrient Mixtures F-12. This formulation combines high concentrations of glucose, amino acids and vitamins with 15% FBS. It is evident that mixture of seeds and proteins (Fig. 3, grey squares) in these conditions do not reach values of fibrils like it was in the assay at acid conditions. This might be due to different factors: first, stability of native proteins at pH of DMEM/F-12 is higher and therefore they have lower affinity towards seeds; then the presence of high concentration of sugars and other proteins might have a partially protective role.

The popular SH-SY5Y neuroblastoma cell *in vitro* system was used as cellular model. SH-SY5Y cells are characterized morphologically by neuroblast-like, non-polarized cell bodies with few, truncated processes. One of the characteristics of SH-SY5Y cells is that cultures include both adherent and floating cells, both types of which are viable. In this work, we utilized only adherent populations and discarded the floating cells during media washing after experimental time ended. Cells were grown in a humidified chamber with 5% CO_2_ at 37°C. Before immunocytochemistry cells were fixed on the surface. Actin filaments were visualized by TRITC-conjugated phalloidin, the nucleus of the cells by DAPI and fibrillar aggregates by ThT.

**Figure 4:**
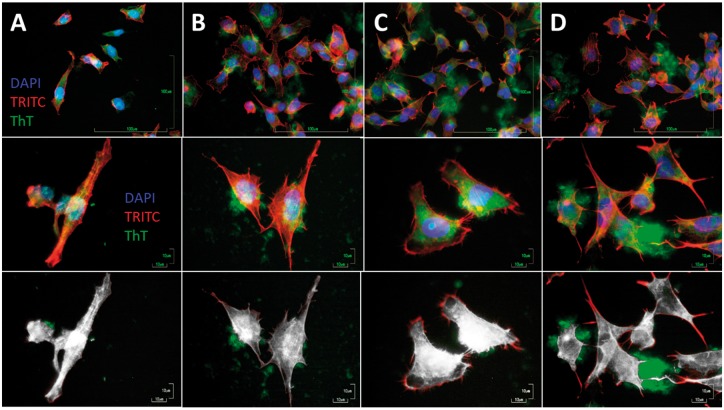
Fluorescence images of SH-SY5Y cells cultured for 48 h in the presence of different lysozyme forms in columns: (**A**) proteins, (**B**) protein with seeds, (**C**) seeds and (**D**) mature fibrils. In upper row low magnification images of superposition of the three dyes used: DAPI (blue), TRITC (red) and ThT (green). In the middle row, high magnification images show typical single cells with accumulation of amyloid assembly nearby, preferential deposition in cell area is evident. Lower row colocalization between two of the dyes (green ThT and red TRITC). Pixel intensity spatial correlation analysis of fibrils and cells was performed to express the intensity of colocalized objects in each component of a dual‐colour image, correlation and co‐occurrence, respectively

Fluorescence images of SH-SY5Y cells and amyloid assemblies are shown in Fig. 4. Cells cultured for 48 h in the presence of A) proteins, B) protein with seeds, C) seeds and D) mature fibrils are reported in both big scale and details. Mild ThT fluorescence is observed even for proteins (Fig. 4 column A) as expected by ThT spectra shown in Supplementary Fig. S1. The images of the single cells show accumulation of amyloid assembly close to the cells. Presence of amyloid assembly exhibited increased reorganization and accumulation of fibres at cell peripheries compared to surroundings. To emphasize this fact, we measured the amount of colocalization between two of the dyes (green ThT and red TRITC) in the images (Fig. 4, bottom raw). Pixel intensity spatial correlation analysis of fibrils and cells was performed to express the intensity of colocalized objects in each component of a dual-colour image, correlation and co-occurrence respectively was calculated using FIJI ImageJ software [16]. White areas indicate a high level of colocalization (above a chosen threshold), while the areas where a single dye is present appear with their own colour.

**Figure 5:**
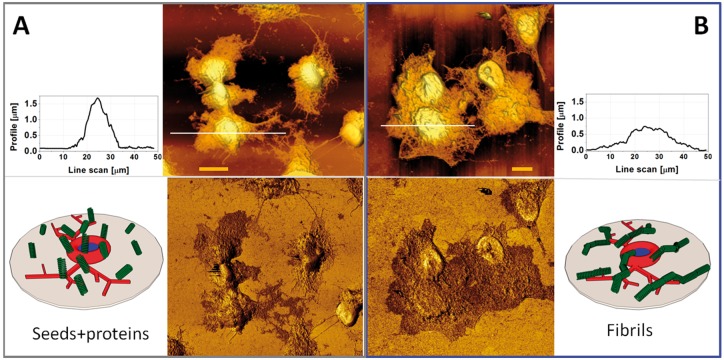
Typical AFM image of SH-SY5Y cell with (**A**) seeds with proteins and (**B**) mature fibrils deposited after 3 h of cell adhesion and grown for 24 h. Fixed cells were imaged in air in semi-contact mode. Height image in upper row with typical line scan profile on respective side shows difference in RMS roughness value for cells exposed to seeds and proteins is 355 (±20) nm, contrariwise in the case with fibrils is 125 (±20) nm. Reason of such difference is suggested in sketch design below by different way of deposition of amyloid assembly. The scale is 10 µm. Adequate phase images in lower row evidence difference in viscoelastic properties of cells (light colour) and amyloids (dark colour)

Samples were than characterized by AFM to get a morphological characterization at the nanoscale in ambient conditions. Typical AFM images of SH-SY5Y cells with amyloid assembly growth for 24 h are shown in Fig. 5. Cells incubated with (A) seeds+proteins and (B) mature fibrils are displayed, in the upper raw the 3D rendering of the topography while in lower one the corresponding phase images. Visualization of the SH-SY5Y with amyloids assemblies shows the typical morphology of cells complex with fibrils (Fig. 5B), in the case for mixture proteins and seeds weaker adhesion and respectively higher profile of cell is observed (Fig. 5A side profile). Root mean squared (RMS) roughness calculated for both cases supported these conclusions. RMS value in the case of fibrils is 125 ± 20 nm, however for seeds and proteins almost three times higher value (355 ± 20 nm) was measured. This could be explained by the different adhesion of seeds onto cell membrane compared to fibrils (Fig. 5).

Phase Imaging is a powerful tool that is sensitive to surface stiffness/softness and adhesion between the tip and surface. It can map different local viscoelastic material properties and provides information about qualitative differences in chemical composition, adhesion and friction.

From the literature, Young’s modulus of neural cell is around 1–10 kPa [17], while for lysozyme molecule/aggregates it is ∼0.2 to 0.5 GPa [18], instead amyloid fibrils are among the stiffest biological materials presently known with a Young’s modulus on the order of 3–20 GPa [19].

Phase images (Fig. 5, lower raw) clearly identify different stiffness of cell nucleus and body (light colour) and amyloid assemblies (dark colour) on the surface, as well as its complex distribution around cell. Preferential localization of amyloid assembly in correspondence of the positions of the cell body is visible as well, this was already observed in our previous study with insulin [20].

From literature [21] we know that lysozyme forms a nontrivial type of amyloid fibrils under controlled denaturation conditions of high temperature and low pH. The reported persistence length ranges between 250 nm and several μm. Since bottom limit value is very similar to the length of our seeds their stiffness could contribute a non-horizontal adsorption on the cell membrane during incubation (Fig. 5A, sketch), the presence of proteins together with seeds can additionally contribute to the interaction with cells membrane. However, long chains of mature fibrils can relax horizontally on the cell body (Fig. 5B, sketch).

**Figure 6:**
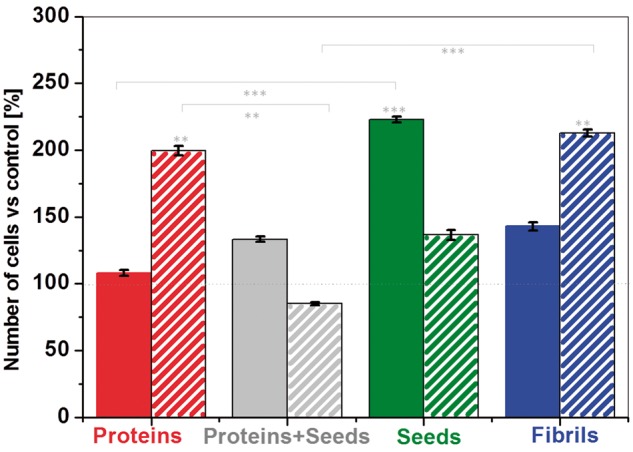
Number of SH-SY5Y cells versus control experiment (100%) after 24 h (solid) and 48 h (dashed) of incubation with different amyloid forms. Cell culture with both native protein (red) and mature fibrils (blue) shows typical proliferation, whereas seeds and mixture of seeds and proteins present a decrease in number of cells. Error bars are standard deviations. One‐way ANOVA test (Kruskal–Wallis with Dunn’s Multiple Comparison Test) shows **P < 0.05 and ***P < 0.001 (*n* = 4)

For many amyloid-forming proteins, it has been found that monomers and full-length fibrils induce limited toxicity to neurons or other cells [22]. Several studies suggested that both non-fibrillar and small fibrillar aggregates have cytotoxic properties. However, there is a lack of detailed reports about the cytotoxic properties. In our study, the number of neural SH-SY5Y cells upon incubation with different amyloid forms was assessed by counting the DAPI-stained nuclei [23]. To quantify cells number, we extract the fraction number of cells normalized to a control sample. In Fig. 6, the percentage of cells–amyloid complexes versus control (100%) is reported. Upon incubation with freshly prepared protein solution (Fig. 6, red bar) we observed at 48 h an almost 100% increase compared to control experiment, the reason could be that this enzyme is known to contribute with different roles in cell growth. Complex of cells with fibrils (Fig. 6, blue bar) did not affect the number of cells. We can see typical exponential phase of cells growth over 48 h of incubation. Therefore, we consider native protein and lysozyme fibrils as non-toxic species. In contrast, seeds (Fig. 6, green bar) and mixture of seeds and proteins (Fig. 6, grey bar) induced an increase in the number of cells upon 24 h incubation followed by a significant decline of cell survival at 48 h. In the case of seeds number of cells drop almost 50% and seeds with protein more than 80%.

## Discussion

There is debate regarding the true pathogenic role of oligomers and mature fibrils in neurodegenerative diseases. Early studies suggested that mature amyloid fibrils were the main pathogenic entities involved in amyloidosis [24]. In contrast, there is equally strong evidence that oligomeric species have also a pathogenic role in these disorders [25]. The perception of the role for amyloid fibrils has changed over recent years and it has been suggested that their true function may be that of an evolutionary protection mechanism employed by the organisms; in this frame it would take place after a major damage carried out at an earlier phase by the soluble oligomers [26]. Our findings are in good agreement with previous study on toxicity of lysozyme oligomers on rat pheochromocytoma (PC12) cells [25], where authors concluded, that native HEWL had no effect on number of the cell and that the aggregated state of the protein is inducing cell death. In the same work sonicated fibrils used to treat cells showed increased apoptotic staining, whereas fibrils generate secondary necrotic, non-apoptotic death.

In conclusion, our findings confirm that innocuous hen lysozyme can be engineered to produce both cytotoxic soluble prefibrillar aggregates and mature amyloid fibrils, further strengthening the claim that supramolecular structure rather that the identity of the protein, is the key of cellular toxicity and of the underlying specific cell death mechanism. Seeds produced by ultrasonication showed higher cytotoxicity, suggesting that, although ultrasonication might be a useful for biomedical applications, potential cytotoxic species are produced. It is possible that the hydrophobic surface exposed by seeds especially at their extremities is responsible for cytotoxicity.

This result is of particular significance in the light of the recent conclusion that the ability to form highly ordered amyloid fibrils is itself a general property of proteins [27]. This toxicity is likely to arise because in these early aggregates hydrophobic side-chains and other regions of the polypeptide chain will be much more accessible than in the fully formed mature fibrils. We report here that sonication of fibrils results in the formation of aggregates that have seeding properties and are toxic to cells. These results have important and far-reaching implications for the use of sonication in medicine and biotechnology.

## Funding

This work was partially supported by Slovak grand agency VEGA 2/0145/17, MVTS COST 083/14 action BM1405, SAS-MOST JRP 2015/5 and CNR-SAS bilateral projects CUP B52F15000340005 and CUP B52I12000320005, Italian flagship NANOMAX, N-CHEM. Microscopy was carried out at the SPM@ISMN facility.

*Conflict of interest statement*. None declared.

## Supplementary Material

Supplementary Figure S1
