# Supplementary material for: Amyloid fragments and their toxicity on neural cells
Source: Regen Biomater. 2019 Mar 11;6(2):121–7. doi: 10.1093/rb/rbz007 (PMC6446995; doi:10.1093/rb/rbz007)
Supplement: Supplementary Figure S1 [file rbz007_supplementary_figure_s1.pdf]

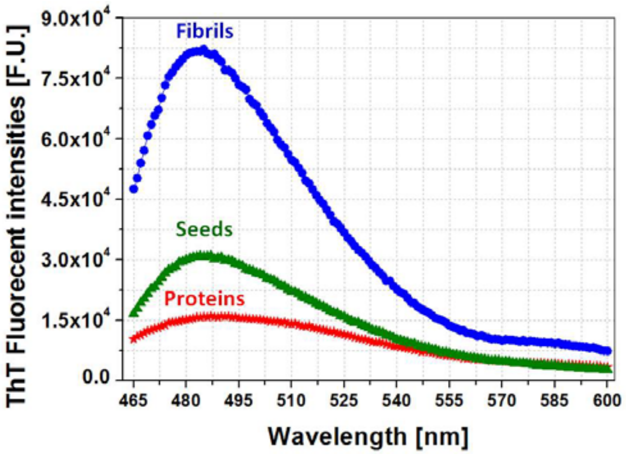

Figure S1 Fluorescence emission spectra of 10 µM native HEWL (red stars), seeds (green triangles) and fibrils (blue circles) upon binding of ThT dye, after 1h incubation in acidic conditions, at 37°C and in dark.
